# Measuring human metacognition in GPT-assisted cognition: development and psychometric validation of a novel scale

**DOI:** 10.3389/fpsyg.2026.1823525

**Published:** 2026-05-28

**Authors:** Mahima Anna Varghese, Poonam Sharma

**Affiliations:** Department of Social Science, School of Social Science and Language, Vellore Institute of Technology, Vellore, India

**Keywords:** artificial intelligence - AI, GenAI, GPT, metacognition, scale development

## Abstract

**Introduction:**

The current age witnessed the development of artificial intelligence and how generative AI tools altered engagement of individuals in their cognitive tasks, which raises important questions about the role of human metacognition in AI-assisted environments. While traditional metacognition theories have extensively examined how people monitor, regulate and evaluate their cognitive processes, it is worth noting that it was for a human only learning context and do not adequately capture metacognitive awareness when cognition is partially shared with AI. The absence of context-specific measurement instruments limits the ability of researchers to systematically investigate how individuals consciously engage with AI during various tasks. Addressing this gap, the present study develops and validates a psychometric instrument designed to measure GPT-assisted metacognitive awareness.

**Methodology:**

Using a multi-stage scale development procedure, items were generated based on classical metacognitive theory and adapted to AI-mediated cognitive contexts. The scale was evaluated through expert content validation, exploratory factor analysis (EFA), confirmatory factor analysis (CFA) and reliability and construct validity assessment using separate samples.

**Results:**

EFA initially identified a three-factor structure consisting of 23 items representing distinct but related dimensions of GPT-assisted metacognitive awareness. However, subsequent CFA and validity assessments indicated substantial overlap between two factors, leading to a refined two-factor model demonstrating superior construct validity and parsimony. The overall scale demonstrated strong internal consistency.

**Discussion:**

The findings suggest that metacognitive awareness in GPT-assisted contexts represents a measurable cognitive construct that extends beyond traditional models of human only metacognition. The proposed scale provides a foundational tool for examining conscious versus passive AI use, understanding cognitive regulation in AI-assisted learning and informing educational and policy discussions surrounding responsible AI integration.

**Conclusion:**

By offering a validated measurement framework, the study contributes to emerging research on human-AI cognitive interaction and metacognitive regulation in the age of generative AI and in turn support SDG Goal 3, 4, and 9.

## Introduction

### Understanding human metacognition

Human metacognition is defined as an individual’s knowledge and management of their own cognitive processes which includes the ability to reflect on what they know, how they learn and how efficiently cognitive techniques are used during task performance (Flavell, 1979; Katyal and Fleming, 2024; Lund and Russell, 2022; Nelson, 1990). Researchers differentiate between local metacognition, which relates to moment-by-moment confidence assessments about certain cognitive tasks and global metacognition, which refers to general self-beliefs about one’s skills and performance across longer periods (8,9). Local metacognitive judgments, such as confidence in a specific answer, might inform global assessments of task or domain performance (10). Metacognition involves monitoring, control (regulation) and metacognitive knowledge and experience (Katyal and Fleming, 2024; Rivers et al., 2020). Monitoring evaluates confidence and correctness of judgments, while control adjusts cognitive processes depending on this evaluation (Lin, 2020).

Metacognition research rose to popularity in the 1970s and 1980s, notably in developmental, educational and neuropsychology, as self-assessment was recognized as important in directing learning (Katyal and Fleming, 2024). Metacognition consists of two interconnected components- metacognitive knowledge (awareness of one’s cognitive abilities, limits and methods) and metacognitive regulation (planning, monitoring and assessing cognitive activity) (Lund and Russell, 2022; Schraw, 1998; Schraw and Dennison, 1994; Schraw and Moshman, 1995). This reflective skill allows people to adjust learning tactics, correct errors and improve performance in academic, professional and everyday problem-solving environments (Schraw, 1998). Metacognition is often recognized as a foundation for higher order cognition and self-directed learning (Flavell, 1979). Advances in measuring techniques have allowed new neuroscientific discoveries in metacognition. Metacognition is important for cognitive control because it allows people to monitor and manage their own cognitive activity. Its failure has been associated to negative results in educational and therapeutic contexts, as well as social coordination (Katyal and Fleming, 2024). Furthermore, research has provided insights into the neurobiologic foundations of metacognitive abnormalities in schizophrenia (Brown et al., 2014).

### Role of human metacognition in learning and performance

Decades of cognitive and educational research have shown that human metacognition is crucial for learning efficiency, information transfer, academic success and professional competence (Fahrni et al., 2025; Teaching + Learning Lab, n.d.). Individuals with higher level of metacognitive awareness tends to think thoroughly, evaluate their outcomes more deeply and modifies it in case of some difficulty (Jiang et al., 2023). These qualities promote deeper comprehension, fewer intellectual mistakes and consistent performance on difficult tasks (MasterSoft, 2024). Metacognition is not limited to an individual’s schooling it also supports their decision-making, creativity and helps in adaptive problem solving in real world settings (Jiang et al., 2023). Due to this, enhancing metacognitive awareness has become a primary objective in educational psychology.

### AI assistants and human metacognition

AI assistants have become widely available in educational, professional and personal settings. Interactive chatbots, writing and coding assistants are increasingly being combined into smartphones, educational apps and search platforms (Labadze et al., 2023). Their minimal entry demands, quick response and numerous capabilities have resulted in a remarkable level of usage (Microsoft, 2025). This rapid growth of artificial intelligence (AI) assistants has changed the environment in which metacognition happens. Unlike traditional learning methods, AI assistants constantly contribute to cognitive tasks by giving explanations and practical suggestions (MasterSoft, 2024). This change raises basic questions about the way human metacognition functions when cognition is mostly distributed to AI assistants (Varghese and Sharma, 2025). While artificial intelligence will assist individuals to think more clearly and reduce their mental load, it could also impact how they check for their understanding, assess accuracy of their information and manage the way how they learn (Gerlich, 2025; Steyvers and Kumar, 2023). Human metacognition in AI-assisted settings differs compared to human metacognition in human only learning contexts (Schneider, 2025). Now AI-assisted thinking is no longer a unique concept but a common cognitive habit particularly among students, professionals and young people.

### Benefits and concerns of AI usage for human metacognition

When talking about the benefits of using AI, it can be seen that AI assistants have several advantages that can aid in improving human metacognition. They can assist users in explaining what they want while planning a task, giving immediate feedback while monitoring their task, and providing review problem identification (Kytes, 2026; Khosravi et al., 2024). When used reflectively AI technology can promote creative thinking and intellectual exploration (Habib et al., 2024; Marrone et al., 2022). However, these benefits come with significant downsides. Too much reliance on AI-generated outputs could restrict self- observation, weaken critical assessment and encourage superficial work involvement (Carobene et al., 2023; Zhai et al., 2024). AI assistants can therefore either improve or decrease metacognitive awareness depending on how they are used.

A rising body of research raises concerns about the negative consequences of excessive or unregulated AI use (Abbas et al., 2024; Battal, 2025). Persistent reliance on AI companions may lead to increased cognitive dependency, less independent thinking, and reduced self-regulation (Kooli et al., 2025). Regularly assigning cognitive tasks to artificial intelligence may eventually limit creativity, problem solving, individuality and cognitive focus (Huang et al., 2024; Kooli et al., 2025). These difficulties are similar to prior arguments about automation and cognitive offloading but here they are further complicated by generative AI’s capacity to replicate reasoning and explanation (Chirayath et al., 2025; Jose et al., 2025; Lee et al., 2025). Understanding when AI usage boosts cognition and when it substitutes critical thinking is thus an important research objective.

### Regulating human metacognition in the era of AI

In the age of AI assisted thinking, managing human metacognition has never been more important. Effective cognitive functioning nowadays involves not only knowledge of one’s own inner thought processes but also a knowledge of external AI systems. In this context, metacognitive control is learning when to depend on AI, when to question its outputs, and when to withdraw in favour of your own thinking (Markauskaite et al., 2022). Without such control while focusing on AI’s cognitive benefits, long-term impact on a person’s individuality and their intellectual growth might get unnoticed.

Traditional metacognitive frameworks have always considered cognition as an internally regulated process involving planning, monitoring and evaluation (Flavell, 1979; Schraw and Dennison, 1994). But the emergence of AI-assisted cognition challenges this internalist perspective. Drawing on distributed cognition theory (Hutchins, 1995), cognitive processes are increasingly understood as being distributed across individuals, tools and environments. Similarly extended mind framework (Clark and Chalmers, 1998) posits that external technologies can function as integral components of cognitive systems rather than merely supporting them. In AI-mediated contexts, users frequently offload cognitive tasks to external tools such as search engines and generative AI systems thus altering cognitive effort, perceived competence and sense of control (Risko and Gilbert, 2016). This shift reflects a transition from internally bounded cognition to a hybrid human-AI cognitive system. At the same time, contemporary research highlights important behavioral implications of such reliance on algorithmic systems. Users may develop a tendency to over-trust AI-generated outputs, often prioritizing them over human judgment, which can influence decision-making processes and metacognitive evaluation (Logg et al., 2019). This introduces a critical extension to traditional metacognitive theory that regulation is no longer limited to internal cognition but must also encompass the monitoring and evaluation of externally generated information. The present study integrates these perspectives by conceptualizing GPT-assisted metacognition as a hybrid system involving both task-oriented engagement with external cognitive tools and reflective regulation of their use, thereby extending classical metacognitive theory into the domain of AI-mediated cognition.

### Significance of assessing metacognition in AI-assisted situations and existing tools

Considering AI’s increasing role in how people think, reliable measurement techniques are essential for comprehending how metacognitive awareness functions in AI-assisted scenarios. Measurement allows researchers to distinguish between conscious AI usage and passive dependence, uncover individual variations in regulatory awareness and assess the cognitive effect of incorporating AI. Without context-specific measuring tools, it is impossible to determine if AI use improves learning or just speeds up job performance at the price of deeper understanding.

Several proven methods for assessing human metacognition have been created, the most notable of which are self-report measures that test metacognitive knowledge and control in typical learning settings (Terneusen et al., 2023). The Metacognitive Awareness Inventory and associated measures have been widely used to investigate the planning, monitoring and assessment processes during learning. However, these instruments were created for situations where cognition is internally controlled and therefore do not take into account the possibility of conversational AI systems that actively affect cognitive processes. As a result, traditional methods may fail to capture important aspects of metacognitive awareness unique to AI-assisted cognition.

### The need for a GPT-specific metacognitive awareness instrument

Despite the widespread usage of artificial intelligence (AI), no validated psychometric tool is presently available to test metacognitive awareness during interactions with GPT-based systems. This is a big gap in the literature. The current study fills this gap by creating and testing a scale that measures how people plan, monitor, manage and evaluate their cognition when using GPTs. Since it is not practical nor desirable to completely avoid AI, the creation of this tool is both necessary and timely. More importantly, improving metacognitive awareness is necessary to guarantee positive and beneficial interactions with artificial intelligence.

### Scope and applications of the proposed scale

The recommended tool can be helpful in a variety of contexts. It can be used to evaluate researchers, educators and student’s metacognitive awareness. This action might support policy discussions on ethical AI use, educational initiatives and digital literacy programs. The tool creates an environment for efficient use of AI while respecting human creativity, autonomy and cognitive development by enabling comprehensive evaluation of metacognitive awareness in GPT-assisted settings.

## Methodology

### Study design

The current study used a cross-sectional psychometric tool development and validation methodology. The major purpose was to create and validate a concept-based self-report tool for assessing an individual’s metacognitive awareness when using GPT. The scale was adapted from the Metacognitive Awareness Inventory by Schraw and Dennison (1994). The initial item pool consisted of 59 items, developed through systematic adaptation of the Metacognitive Awareness Inventory (MAI) to reflect AI-mediated cognitive contexts. All items were grounded in the core dimensions of metacognitive knowledge and regulation, while being reworded to explicitly incorporate interaction with GPT systems. In addition to adapting existing MAI items, the scale incorporated a conceptual emphasis on trust in GPT outputs, including aspects such as reliance, verification, and perceived credibility of AI-generated information. This was necessary to capture dimensions of metacognitive awareness that emerge uniquely in AI-assisted environments. The scientific procedure adhered to globally approved principles for the construction of psychological scales with a focus on rigorous reliability and validity testing.

Item development, expert content validation, exploratory factor analysis (EFA), confirmatory factor analysis (CFA) and reliability and construct validity evaluation comprised the multi-stage validation approach that was used. EFA and CFA were performed on separate samples to improve generalizability.

### Construct definition and conceptual framework

While the scale’s core structure was inspired by Schraw and Dennison’s (1994) Metacognitive Awareness Inventory (MAI) (Schraw, 1998; Schraw and Dennison, 1994; Schraw and Moshman, 1995), the current instrument reflects a relevant restructuring of metacognitive awareness for AI-mediated cognitive contexts, i.e., conceptualizing GPT-assisted metacognitive awareness as a context-specific extension of traditional metacognition. Classical metacognition is described as knowledge of cognition and its management. However, GPT-assisted situations bring fundamentally novel cognitive circumstances, such as instant interactive responses, fast response improvement and partial allocation of intellectual work. These processes are partially externalized, as individuals interact with generative systems that actively contribute to cognitive tasks. This construct captures how individuals plan their interactions with GPT, monitor the accuracy and usefulness of AI-generated outputs, regulate strategy use in response to system feedback and evaluate their own understanding and performance in AI-assisted tasks.

The scale’s theoretical foundation focuses on metacognition theory, self-regulated learning and human-AI interaction research showing GPT as a thinking companion that changes the structure of metacognitive control. It sees metacognitive awareness as a hidden and complex phenomenon that shows itself through observable voluntary behaviours and personal evaluations while using GPTs. The final scale aims to represent meaningful individual differences in how users engage both consciously and subconsciously with GPT systems. All questions were given as first-person statements and response in the form of True/False.

### Content validity

To establish content validity, a panel of subject matter experts with backgrounds in clinical psychology and technology evaluated the preliminary question pool. Experts assessed each item separately for conceptual relevance and clarity to the presented concept. Items with an issue were modified for grammatical accuracy and conceptual overlapping was decreased and theoretically weak items were adjusted based on expert feedback. The modified items demonstrated an excellent fit with the construct definition and adequately covered the conceptual domain.

### Participants

Participants between the age range of 18 to 25 and those who were familiar with the aspect of artificial intelligence were considered for the study. Individuals who were not aware about AI-based applications were removed to ensure appropriate responses. A purposive sample strategy was used with online recruiting tools. In order to ensure psychometric accuracy, a sample of 136 participants were collected before conducting the exploratory factor analysis and a sample of 959 were taken before conducting the confirmatory factor analysis. The final sample size is higher than the frequently suggested minimum for factor analysis, allowing for stable parameter estimates and accurate model testing. Data were acquired via an online self-administered questionnaire. Participants were given an information sheet that explained the goal of the study, the voluntary nature of participation, the confidentiality of replies and the estimated completion time. Prior to participation, participants provided informed consent electronically. No personally identifying information was gathered. Before being analyzed, responses were checked for completeness. The sample primarily consisted of undergraduate and postgraduate Indian students from diverse academic disciplines. In terms of GPT usage, the sample had adequate familiarity with AI-based tools, making it appropriate for examining GPT-assisted metacognitive awareness (Table 1).

**Table 1:** Demographics of participants.

Variable	Category	Exploratory	Confirmatory factor analysis
Gender	Male	85	491
	Female	51	468
	Other	—	—
Age	Mean (SD)	19	19
	Range	18–25	18–25
GPT usage frequency	Daily	102	231
	Weekly	24	252
	Monthly	4	66
	Rarely	6	410

### Statistical analysis

All statistical analyses were conducted using IBM SPSS Statistics and JMP software. Preliminary data screening, descriptive statistics, reliability analysis, and exploratory factor analysis (EFA) were performed using SPSS. Confirmatory factor analysis (CFA) was conducted using JMP. An exploratory factor analysis was performed to determine the scale’s underlying latent structure. Principal axis factoring was used since the data was not believed to be normally distributed and as the purpose was to find latent constructs rather than improve explained variance. An oblique rotation approach was adopted and sample adequacy was assessed using the Kaiser–Meyer–Olkin (KMO) measure and Bartlett’s test of sphericity. Factor retention was determined using a combination of eigenvalues higher than one, scree plot examination and theoretical interpretability. During exploratory factor analysis, items were further evaluated using standard psychometric criteria. Items with factor loadings below 0.30 and those exhibiting cross-loadings above 0.30 across multiple factors were removed. However, a small number of theoretically important items, particularly those related to trust in GPT, were retained despite marginal statistical values to preserve conceptual completeness. The final factor structure reflects the empirical reorganization of MAI-based dimensions into constructs specific to GPT-assisted metacognition.

Confirmatory factor analysis was used to validate the factor structure discovered during EFA. The model fit was tested using numerous complementing indicators, such as the comparative fit index (CFI), Tucker-Lewis index (TLI), root mean square error of approximation (RMSEA) and standardized root mean square residual (SRMR). Internal consistency reliability was assessed using both Cronbach’s alpha and McDonald’s omega.

### Construct validity

Standardized factor loadings, average variance extracted (AVE) and composite reliability were used to assess convergent validity. Discriminant validity was assessed using both standard variance-based criteria and the heterotrait- monotrait correlation ratio (HTMT).

### Ethical considerations

The study followed ethical guidelines for research employing human subjects. Participation was entirely voluntary and informed consent was given before data collection. Individuals could withdraw at any moment without any consequence. Data was securely saved and utilized only for research reasons.

## Results

### Preliminary item analysis

The preliminary version of the adapted scale had 59 items in order to capture various aspects of GPT-assisted metacognitive awareness and regulation. The initial reliability analysis showed strong internal consistency (Cronbach’s *α* = 0.889), indicating that the item pool was widely reliable. However, examination of corrected item-total correlations indicated that few items contributed barely to the overall scale. Items with item-total correlations less than 0.20 and those with increased Cronbach’s alpha after removal were excluded due to their poor discrimination. Theoretically significant items related to trust in GPT were retained despite low item-total correlation to analyze their contribution to the latent structure, resulting in a 47-item version with a good reliability (*α* = 0.890).

### Exploratory factor analysis

The KMO and Bartlett tests confirmed the suitability of data for factor analysis. The KMO value (KMO = 0.647) and Bartlett value (*χ*^2^ = 2236.76, *df* = 1,081, *p* < 0.001) was considered adequate. The 47-item initial questionnaire underwent exploratory factor analysis (EFA) with Principal Axis Factoring and direct oblimin rotation.

Initial extraction and parallel analysis revealed a three-factor structure. However, the analysis revealed numerous items with low primary loadings (<0.30) and high cross-loading. These items were systematically eliminated with EFA updated after each removal. As items were removed the factor structure grew more stable. EFA produced a 23-item scale that properly loaded into three clear factors with no significant cross-loading. The three variables indicated separate but interconnected features of GPT-assisted metacognitive awareness.

**Figure 1:**
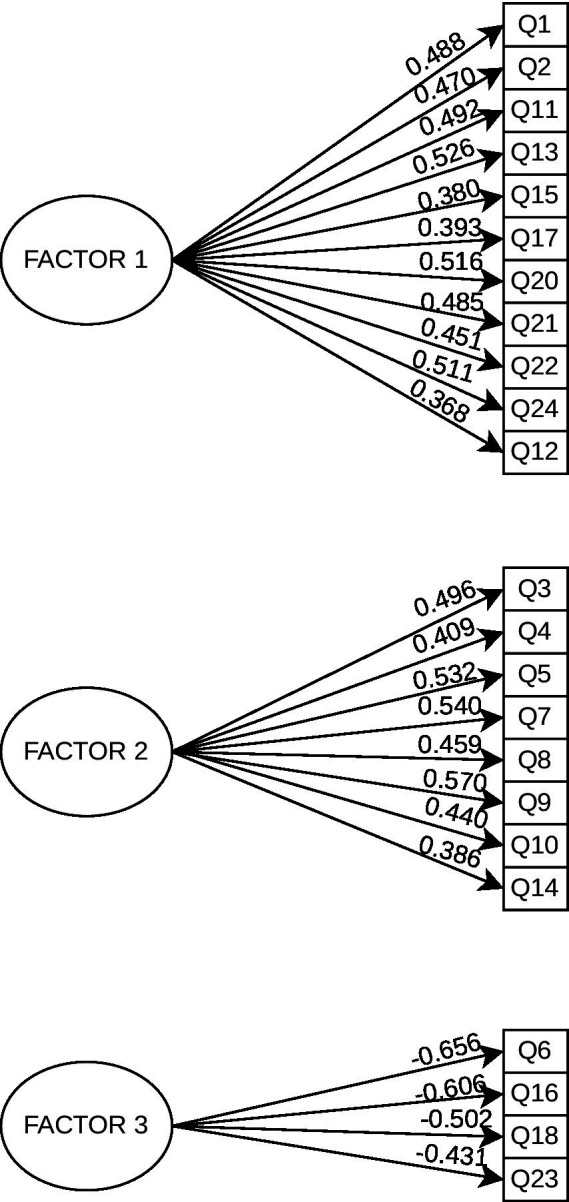
The three factors and their factor loading from EFA.

The 23-item scale has strong internal consistency (*α* = 0.802) indicating acceptable reliability for a new instrument. Eliminating any item did not significantly boost subscale reliability implying that all remaining items provided significant benefits to their own domains (see Figure 1).

### Confirmatory factor analysis

Confirmatory factor analysis (CFA) was carried out to confirm the three-factor structure found by EFA. The Comparative Fit Index (CFI) was just below the conventional standards (CFI: 0.824) in the first model estimation indicating an unsatisfactory fit. Further examination revealed very high inter-factor correlations, particularly between Factor 2 and Factor 3, indicating lack of discriminant validity. A revised two-factor model was tested, combining conceptually overlapping dimensions. It was also found that Item Q24 showed significant cross-loadings between Factors 2 and 3 having modification indices above 15 showing structural ambiguity. As a result, this item was eliminated. The two-factor model demonstrated improved parsimony and acceptable model fit.

The fit indices for the improved CFA model suggested good overall fit. Fit indices indicated strong model (*χ*^2^ = 432.89, *df* = 208, *p* < 0.001), with CFI = 0.912, TLI = 0.902, SRMR = 0.034 and RMSEA = 0.034 (90% CI [0.029, 0.038]). These results show minimal residual error and great incremental fit, meeting or surpassing widely recognized standards for good model fit. These findings validate the two-factor measurement model’s factorial validity (Table 2).

**Table 2:** Factor loading estimates for items based on CFA.

Item	Question	Stand. loading (*λ*)	SE	z-value
Factor 1: strategic & motivational engagement with GPT				
Q1	I manage my time effectively with GPT to ensure I have enough time to complete my tasks.	0.442	0.031	14.327
Q11	GPT often serves as motivation to help me complete my tasks.	0.467	0.030	15.442
Q12	In some cases, I feel confident in GPT’s information and do not verify its accuracy.	0.318	0.034	9.441
Q14	While using GPT, I find myself pausing regularly to check my comprehension.	0.421	0.031	13.445
Q15	I find GPT responses more detailed and better than the ones which I obtain from other sources.	0.465	0.030	15.325
Q17	GPT regularly helps to create better organizational structure of text which helps me to learn better.	0.512	0.029	17.679
Q20	I generally organize my time effectively with the help of GPT to accomplish my goals.	0.534	0.028	18.797
Q21	I tend to learn more with the help of GPT when I am interested in the topic.	0.571	0.027	21.078
Q22	I typically ask myself questions about my progress while learning something new with GPT.	0.453	0.031	14.842
Factor 2: metacognitive regulation and reflective processes				
Q6	I usually try using different prompts with GPT Chatbots depending on the nature of the task.	0.426	0.031	13.637
Q16	I frequently change strategies when I fail to understand GPT information.	0.379	0.032	11.656
Q18	Ordinarily, I input instructions carefully to GPT Chatbots before I begin a task.	0.423	0.031	13.518
Q23	Usually, I stop and reread GPT-given information when I get confused.	0.356	0.033	10.806
Q13	I frequently find myself using helpful learning strategies automatically when I use GPT.	0.509	0.029	17.585
Q8	I normally have control over how well I learn with the help of GPT.	0.352	0.033	10.671
Q2	I understand the intellectual strengths of various GPT chatbots.	0.372	0.033	11.448
Q3	Before starting a task, I think about what I really need to learn from using the GPTs.	0.431	0.031	13.849
Q4	I can usually identify which information provided by GPT is important and which is less significant.	0.426	0.031	13.619
Q5	I often ask myself if I have considered all options, including those suggested by GPT, before solving a problem.	0.399	0.032	12.512
Q7	After completing a task with GPT’s help, I regularly reflect on whether there was a simpler way to accomplish it.	0.352	0.033	10.676
Q9	I periodically review GPT-given information for understanding its authenticity.	0.457	0.031	14.958
Q10	I frequently ask myself questions related to the task before I begin using GPT.	0.370	0.033	11.360

All factor loadings were statistically significant at *p* < 0.001. Lower loadings were retained due to theoretical relevance and exploratory nature of the construct.

### Instrument reliability

Internal consistency reliability was evaluated for each factor and for the overall instrument. McDonald’s omega (*ω*) was given priority due to its suitability for multidimensional congeneric models (65) For Factor 1, reliability was acceptable (*ω* = 0.714; *α* = 0.709) and for Factor 2, reliability was acceptable (*ω* = 0.718; *α* = 0.716) the overall reliability of the 22-item instrument was strong (*ω* = 0.814; *α* = 0.811), indicating satisfactory internal consistency at the scale level. According to Loewenthal, a slightly lower index is acceptable in case (1) there is good evidence for validity, (2) there are good theoretical reasons for the scale operationalisation, and (3) when the scale is relatively short (less than about 10 items). Furthermore, inspection of the “*α*/*ω* if item deleted” output showed that removing any item did not improve reliability, suggesting that all items contribute meaningfully to their respective latent domains. The scale showed adequate model fit, stable factor structure and satisfactory internal consistency after a thorough process of item purification, exploratory analysis and confirmatory validation. The results validate the application of the tool to evaluate GPT-assisted metacognitive awareness (see Table 3).

**Table 3:** The item analysis.

Item	Cronbach’s alpha if item is deleted	Corrected item total correlations
Q1	0.804	0.374
Q11	0.804	0.377
Q12	0.809	0.265
Q14	0.804	0.366
Q15	0.804	0.375
Q17	0.801	0.429
Q20	0.800	0.445
Q21	0.799	0.476
Q22	0.802	0.400
Q6	0.804	0.361
Q16	0.807	0.317
Q18	0.805	0.356
Q23	0.806	0.321
Q13	0.800	0.444
Q8	0.807	0.313
Q2	0.806	0.318
Q3	0.803	0.381
Q4	0.805	0.358
Q5	0.805	0.344
Q7	0.807	0.309
Q9	0.804	0.376
Q10	0.807	0.310

### Convergent validity and discriminant validity

Composite reliability (CR) and average variance extracted (AVE) which were obtained from the final CFA model’s standardized factor loadings were used to assess convergent validity (Table 4). The two factors had composite reliability values 0.714 and 0.717, respectively, and the scale as a whole showed strong reliability (CR = 0.81). In the scenario of brief multidimensional instruments in particular, these values satisfy acceptable levels for recently established and conceptually separate subscales (Hair et al., 2011). AVE readings were below the standard criteria, ranging from 0.165 to 0.221. However, given all standardized factor loadings were statistically significant (*p* < 0.001), composite reliability values were higher than the minimum permissible limits and the overall measurement model showed good fit, convergent validity was deemed appropriate (Fornell and Larcker, 1981; Little et al., 1999).

**Table 4:** The AVE and CR of each factor.

Factor	AVE	CR
Factor 1	0.221	0.714
Factor 2	0.165	0.717

The heterotrait-monotrait ratio of correlations (HTMT) (Table 5) and inter-factor correlations (Table 6) obtained from the CFA model were used to evaluate discriminant validity. With values below the suggested cutoff of 0.85, HTMT values showed good discriminant validity across the factor pairs. This degree of linkage is deemed appropriate given the developing context of GPT-assisted cognition and the conceptually related nature of metacognitive processes. The two-factor model did better than other alternatives suggesting keeping of conceptually related but clearly separate elements. These findings provide strong proof that the proposed two-factor structure is discriminately valid.

**Table 5:** Discriminant validity (HTMT).

Factor pair	HTMT	Interpretation
Factor 1–factor 2	0.78	Good

**Table 6:** Inter-factor correlations.

Factor	1	2
1. Factor 1	—	0.799
2. Factor 2	0.799	—

## Discussion

The current work sought to develop and test a psychometric instrument capable of evaluating human metacognitive awareness in GPT-assisted settings, an area where no particular assessment tools are currently available. The results suggests that metacognitive awareness in an environment with GPT support is a contextually extended form of traditional metacognition, rather than a completely separate phenomenon (Terneusen et al., 2023; Tezer, 2025). The presence of interlinked but distinct factors in the scale reveals that GPT is not just a mere information source but it acts as an active thinking companion (Thao et al., 2025; Zhang, 2025). This highlights the relevance of developing context-specific measures of metacognitive awareness of an individual which is applicable in the present world as the traditional models may not fully capture certain aspects.

### Interpretation of the two-factor structure of GPT-assisted human metacognitive awareness

The two factors extracted through the process of Exploratory and Confirmatory Factor analysis gives idea onto the elements influencing metacognitive awareness while using GPT. Even though these factors are conceptually distinct but strongly related throughout the interaction with Artificial intelligence. The moderate correlation between the two factors reveals the conceptual connection in the metacognitive awareness. In spite of the observed connection between the factors, the decision to retain every factor was justified with the improved model fit. It can also be justified conceptually due to the need to discriminate metacognitive awareness and metacognitive regulation, failing which significant individual differences in how the users engage with various GPT platforms would be lost. It is worth noting that metacognitive awareness while interacting with GPT platforms is different from traditional models of metacognitive awareness due to the continuous interaction with AI platforms which provides instant feedback. This finding suggests potential extensions to traditional models of human metacognition and supports to some extent the modern theories which views metacognition as a dynamic and changing system, especially due to the active participation of artificial intelligence in the current educational setting.

The final two-factor structure demonstrates distinct yet related dimensions of GPT-assisted metacognition. Factor 1, labelled as “Task-Oriented Metacognitive Engagement with GPT” captures performance-focused interactions with GPT including efficient task management, motivation, organization of work and perceived enhancement in output quality. The items loading on this factor reflect how individuals utilize GPT as a cognitive tool to facilitate task execution, improve productivity and support goal-directed learning processes. In contrast, Factor 2 labelled as “Metacognitive Regulation and Reflective Processes,” represents higher-order self-regulatory mechanisms involved in planning, monitoring and evaluating one’s cognition during GPT usage. This factor includes behaviours such as adapting strategies, critically reviewing information, reflecting on task performance and questioning the validity and relevance of GPT-generated outputs. Together, the two factors capture both the functional and reflective dimensions of GPT-assisted cognition, supporting the theoretical distinction between task-oriented engagement and metacognitive regulation while maintaining conceptual relatedness between the constructs.

### Psychometric properties as indicators of construct novelty

The psychometric properties provide initial support for the proposed construct which is GPT- Assisted metacognitive awareness. The total scale indicated high reliability when compared to the composite reliability of the subdomains which were adequate for newly constructed scales. This trend implies that whereas individuals may differ significantly in the way certain metacognitive techniques appear throughout GPT usage the overall concept of metacognitive awareness is consistent at the scale level.

Similarly, the average variance extracted (AVE) values were below the standard benchmark. However low AVE values are not surprising in the setting of metacognitive awareness, especially when mediated by AI. This was justified with the HTMT values which supported good discriminant validity in spite of the low AVE Values. Items on the scale were carefully chosen to represent unique aspects of awareness, such as introspective monitoring, strategic adaptation and evaluative judgment. In early-stage construct formation, particularly for complex cognitive phenomena, significant factor loadings, acceptable CR values and acceptable model fit all of which observed in the current study are more relevant indicators of convergent validity. GPT interaction may blur the boundaries between planning, monitoring and evaluation by allowing for quick responses and constant output modification. Users frequently assess comprehension while creating prompts, examine accuracy while getting feedback and adjust approaches during execution of the task rather than post completion.

The psychometric findings suggest that GPT-assisted metacognitive awareness may represent a developing and adaptive concept which is still influenced by the demands of the task, competence of the user and environmental restriction and cannot be said as of now as a consistent behavioural pattern.

### Implications for human-AI interaction and educational practice

When seen from a theoretical aspect, these findings extend existing perspectives on metacognition of metacognition to cover the current scenario. The concept of GPT- assisted metacognitive awareness includes not just one’s own internal thinking but also how those thoughts are motivated when paired with an all-time companion which could influence an individual’s thinking, decision making and other cognitive aspects. From an application point of view, this instrument may serve as a useful tool for understanding an individual’s GPT Usage and to understand whether it’s conscious or passive usage of GPT. These are aspects of the construct which cannot be understood by merely checking the frequency of AI Use. Understanding the pattern of a person’s GPT use is crucial in the present world where concerns regarding cognitive offloading and lack of self- regulation are increasing day by day. The instrument can understand the characteristics of individual with high metacognitive awareness while using GPT by analysing the manner in which they monitor themselves and modify strategies while using GPT and those who rely on GPT without even reviewing them. This distinction may play an important role by giving insights in curriculum development and providing criteria for proper AI-assisted learning. These findings should be interpreted in light of the exploratory nature of the study and the context-specific characteristics of the sample.

### Limitations and directions for future research

In spite of its positive aspects, the study has few limitations. The cross-sectional nature of the study prevents the observation of how GPT assisted metacognitive awareness changes over time. Future researches could assess the longitudinal aspect of this study and determine whether frequent reliance on AI increases or decreases metacognitive awareness and whether it affects others aspects of cognition. The sample primarily consisted of young adults (18–25), educated individuals from a single cultural context, which may limit the generalizability of the findings to broader populations. The use of purposive sampling may also introduce selection bias. Future research should validate the scale across more diverse populations and employ larger, more representative samples. Studies could also examine the structure of GPT-assisted metacognition using hierarchical or bifactor models to determine whether the construct can also be represented as a higher-order latent system. Further studies can also be done to investigate task- specific variations of this instrument. Combining these self- report measures with behavioural data could strengthen the validity of the given construct.

## Conclusion

In this work, a sound psychometric method for assessing metacognitive awareness in GPT-assisted conditions is introduced. The results show a stable factor structure, acceptable reliability and good model fit supporting GPT-assisted metacognitive awareness as a real and measurable concept. By measuring how people use their cognition when interacting with generative AI systems the scale advances both theory and practice. This established the foundation for further studies on learning and cognition in the era of artificial intelligence.

## Data Availability

The raw data supporting the conclusions of this article will be made available by the authors, without undue reservation.
